# Supplementary material for: OpenMM 7: Rapid development of high performance algorithms for molecular dynamics
Source: PLoS Comput Biol. 2017 Jul 26;13(7):e1005659. doi: 10.1371/journal.pcbi.1005659 (PMC5549999; doi:10.1371/journal.pcbi.1005659)
Supplement: S1 Source Code — (ZIP) [file pcbi.1005659.s002.zip › OpenMM-7.0.1-Source/docs/api-python/generated/simtk.openmm.openmm.CustomAngleForce.html]

CustomAngleForce — OpenMM 7.0.1.dev-5e86c4f documentation


# CustomAngleForce¶

*class* `simtk.openmm.openmm.``CustomAngleForce`(*\*args*)¶
:   This class implements interactions between sets of three particles that depend on the angle between them. Unlike HarmonicAngleForce, the functional form of the interaction is completely customizable, and may involve arbitrary algebraic expressions. In addition to the angle formed by the particles, it may depend on arbitrary global and per-angle parameters.

    To use this class, create a CustomAngleForce object, passing an algebraic expression to the constructor that defines the interaction energy between each set of particles. The expression may depend on theta, the angle formed by the particles, as well as on any parameters you choose. Then call addPerAngleParameter() to define per-angle parameters, and addGlobalParameter() to define global parameters. The values of per-angle parameters are specified as part of the system definition, while values of global parameters may be modified during a simulation by calling Context::setParameter(). Finally, call addAngle() once for each angle. After an angle has been added, you can modify its parameters by calling setAngleParameters(). This will have no effect on Contexts that already exist unless you call updateParametersInContext().

    As an example, the following code creates a CustomAngleForce that implements a harmonic potential:

    ```
    CustomAngleForce* force = new CustomAngleForce("0.5*k*(theta-theta0)^2");
    ```

    This force depends on two parameters: the spring constant k and equilibrium angle theta0. The following code defines these parameters:

    ```
    force->addPerAngleParameter("k");
    force->addPerAngleParameter("theta0");
    ```

    Expressions may involve the operators + (add), - (subtract), \* (multiply), / (divide), and ^ (power), and the following functions: sqrt, exp, log, sin, cos, sec, csc, tan, cot, asin, acos, atan, sinh, cosh, tanh, erf, erfc, min, max, abs, floor, ceil, step, delta, select. All trigonometric functions are defined in radians, and log is the natural logarithm. step(x) = 0 if x is less than 0, 1 otherwise. delta(x) = 1 if x is 0, 0 otherwise. select(x,y,z) = z if x = 0, y otherwise.

    `__init__`(*self*, *energy*) → CustomAngleForce¶
    :   \_\_init\_\_(self, other) -> CustomAngleForce

        Create a CustomAngleForce.

        |  |  |
        | --- | --- |
        | Parameters: | **energy** (*string*) – an algebraic expression giving the interaction energy between three particles as a function of theta, the angle between them |

    Methods

    |  |  |
    | --- | --- |
    | `__init__`((self, energy) -> CustomAngleForce) | \_\_init\_\_(self, other) -> CustomAngleForce |
    | `addAngle`((self, particle1, particle2, ...) | addAngle(self, particle1, particle2, particle3) -> int |
    | `addGlobalParameter`((self, name, ...) | Add a new global parameter that the interaction may depend on. |
    | `addPerAngleParameter`((self, name) -> int) | Add a new per-angle parameter that the interaction may depend on. |
    | `getAngleParameters`(self, index) | Get the force field parameters for an angle term. |
    | `getEnergyFunction`((self) -> std::string const &) | Get the algebraic expression that gives the interaction energy for each angle |
    | `getForceGroup`((self) -> int) | Get the force group this Force belongs to. |
    | `getGlobalParameterDefaultValue`((self, ...) | Get the default value of a global parameter. |
    | `getGlobalParameterName`((self, ...) | Get the name of a global parameter. |
    | `getNumAngles`((self) -> int) | Get the number of angles for which force field parameters have been defined. |
    | `getNumGlobalParameters`((self) -> int) | Get the number of global parameters that the interaction depends on. |
    | `getNumPerAngleParameters`((self) -> int) | Get the number of per-angle parameters that the interaction depends on. |
    | `getPerAngleParameterName`((self, ...) | Get the name of a per-angle parameter. |
    | `setAngleParameters`(self, index, particle1, ...) | setAngleParameters(self, index, particle1, particle2, particle3) |
    | `setEnergyFunction`(self, energy) | Set the algebraic expression that gives the interaction energy for each angle |
    | `setForceGroup`(self, group) | Set the force group this Force belongs to. |
    | `setGlobalParameterDefaultValue`(self, index, ...) | Set the default value of a global parameter. |
    | `setGlobalParameterName`(self, index, name) | Set the name of a global parameter. |
    | `setPerAngleParameterName`(self, index, name) | Set the name of a per-angle parameter. |
    | `updateParametersInContext`(self, context) | Update the per-angle parameters in a Context to match those stored in this Force object. |
    | `usesPeriodicBoundaryConditions`((self) -> bool) | Returns whether or not this force makes use of periodic boundary conditions. |

    `getNumAngles`(*self*) → int¶
    :   Get the number of angles for which force field parameters have been defined.

    `getNumPerAngleParameters`(*self*) → int¶
    :   Get the number of per-angle parameters that the interaction depends on.

    `getNumGlobalParameters`(*self*) → int¶
    :   Get the number of global parameters that the interaction depends on.

    `getEnergyFunction`(*self*) → std::string const &¶
    :   Get the algebraic expression that gives the interaction energy for each angle

    `setEnergyFunction`(*self*, *energy*)¶
    :   Set the algebraic expression that gives the interaction energy for each angle

    `addPerAngleParameter`(*self*, *name*) → int¶
    :   Add a new per-angle parameter that the interaction may depend on.

        |  |  |
        | --- | --- |
        | Parameters: | **name** (*string*) – the name of the parameter |
        | Returns: | the index of the parameter that was added |
        | Return type: | int |

    `getPerAngleParameterName`(*self*, *index*) → std::string const &¶
    :   Get the name of a per-angle parameter.

        |  |  |
        | --- | --- |
        | Parameters: | **index** (*int*) – the index of the parameter for which to get the name |
        | Returns: | the parameter name |
        | Return type: | string |

    `setPerAngleParameterName`(*self*, *index*, *name*)¶
    :   Set the name of a per-angle parameter.

        |  |  |
        | --- | --- |
        | Parameters: | - **index** (*int*) – the index of the parameter for which to set the name - **name** (*string*) – the name of the parameter |

    `addGlobalParameter`(*self*, *name*, *defaultValue*) → int¶
    :   Add a new global parameter that the interaction may depend on.

        |  |  |
        | --- | --- |
        | Parameters: | - **name** (*string*) – the name of the parameter - **defaultValue** (*double*) – the default value of the parameter |
        | Returns: | the index of the parameter that was added |
        | Return type: | int |

    `getGlobalParameterName`(*self*, *index*) → std::string const &¶
    :   Get the name of a global parameter.

        |  |  |
        | --- | --- |
        | Parameters: | **index** (*int*) – the index of the parameter for which to get the name |
        | Returns: | the parameter name |
        | Return type: | string |

    `setGlobalParameterName`(*self*, *index*, *name*)¶
    :   Set the name of a global parameter.

        |  |  |
        | --- | --- |
        | Parameters: | - **index** (*int*) – the index of the parameter for which to set the name - **name** (*string*) – the name of the parameter |

    `getGlobalParameterDefaultValue`(*self*, *index*) → double¶
    :   Get the default value of a global parameter.

        |  |  |
        | --- | --- |
        | Parameters: | **index** (*int*) – the index of the parameter for which to get the default value |
        | Returns: | the parameter default value |
        | Return type: | double |

    `setGlobalParameterDefaultValue`(*self*, *index*, *defaultValue*)¶
    :   Set the default value of a global parameter.

        |  |  |
        | --- | --- |
        | Parameters: | - **index** (*int*) – the index of the parameter for which to set the default value - **defaultValue** (*double*) – the default value of the parameter |

    `addAngle`(*self*, *particle1*, *particle2*, *particle3*, *parameters*) → int¶
    :   addAngle(self, particle1, particle2, particle3) -> int

        Add an angle term to the force field.

        |  |  |
        | --- | --- |
        | Parameters: | - **particle1** (*int*) – the index of the first particle connected by the angle - **particle2** (*int*) – the index of the second particle connected by the angle - **particle3** (*int*) – the index of the third particle connected by the angle - **parameters** (*vector< double >*) – the list of parameters for the new angle |
        | Returns: | the index of the angle that was added |
        | Return type: | int |

    `getAngleParameters`(*self*, *index*)¶
    :   Get the force field parameters for an angle term.

        |  |  |
        | --- | --- |
        | Parameters: | **index** (*int*) – the index of the angle for which to get parameters |
        | Returns: | - **particle1** (*int*) –   the index of the first particle connected by the angle - **particle2** (*int*) –   the index of the second particle connected by the angle - **particle3** (*int*) –   the index of the third particle connected by the angle - **parameters** (*vector< double >*) –   the list of parameters for the angle |

    `setAngleParameters`(*self*, *index*, *particle1*, *particle2*, *particle3*, *parameters*)¶
    :   setAngleParameters(self, index, particle1, particle2, particle3)

        Set the force field parameters for an angle term.

        |  |  |
        | --- | --- |
        | Parameters: | - **index** (*int*) – the index of the angle for which to set parameters - **particle1** (*int*) – the index of the first particle connected by the angle - **particle2** (*int*) – the index of the second particle connected by the angle - **particle3** (*int*) – the index of the third particle connected by the angle - **parameters** (*vector< double >*) – the list of parameters for the angle |

    `updateParametersInContext`(*self*, *context*)¶
    :   Update the per-angle parameters in a Context to match those stored in this Force object. This method provides an efficient method to update certain parameters in an existing Context without needing to reinitialize it. Simply call setAngleParameters() to modify this object’s parameters, then call updateParametersInContext() to copy them over to the Context.

        This method has several limitations. The only information it updates is the values of per-angle parameters. All other aspects of the Force (such as the energy function) are unaffected and can only be changed by reinitializing the Context. The set of particles involved in a angle cannot be changed, nor can new angles be added.

    `usesPeriodicBoundaryConditions`(*self*) → bool¶
    :   Returns whether or not this force makes use of periodic boundary conditions.

        |  |  |
        | --- | --- |
        | Returns: | false |
        | Return type: | bool |

    `__delattr__`¶
    :   x.\_\_delattr\_\_(‘name’) <==> del x.name

    `__format__`()¶
    :   default object formatter

    `__getattribute__`¶
    :   x.\_\_getattribute\_\_(‘name’) <==> x.name

    `__hash__`¶

    `__reduce__`()¶
    :   helper for pickle

    `__reduce_ex__`()¶
    :   helper for pickle

    `__sizeof__`() → int¶
    :   size of object in memory, in bytes

    `__str__`¶

    `getForceGroup`(*self*) → int¶
    :   Get the force group this Force belongs to.

    `setForceGroup`(*self*, *group*)¶
    :   Set the force group this Force belongs to.

        |  |  |
        | --- | --- |
        | Parameters: | **group** (*int*) – the group index. Legal values are between 0 and 31 (inclusive). |

High performance molecular simulation on GPUs


### Navigation

- Application Layer
- Library Layer
  - Core Objects
  - Forces
  - Integrators
  - Extras

©2015, Stanford University and the Authors.
|
Powered by Sphinx 1.3.1
& Alabaster 0.7.7
|
Page source
